# Early Allograft Dysfunction After Liver Transplantation: Impact on Clinical Outcomes and Associated Risk Factors

**DOI:** 10.3390/medicina61091710

**Published:** 2025-09-19

**Authors:** Jungho Shin, Suk-Won Suh

**Affiliations:** 1 Department of Internal Medicine, College of Medicine, Chung-Ang University, Seoul 06973, Republic of Korea; junghoshin@cau.ac.kr; 2 Department of Surgery, College of Medicine, Chung-Ang University, Seoul 06793, Republic of Korea

**Keywords:** early allograft dysfunction, liver transplantation, graft survival, transplant complications

## Abstract

*Background and Objectives*: Early allograft dysfunction (EAD), defined as suboptimal initial graft function following liver transplantation (LT), is a serious complication associated with increased post-LT morbidity and mortality. This study aimed to evaluate the impact of EAD on clinical outcomes and to identify associated risk factors. *Materials and Methods*: Ninety-three patients who underwent LT between July 2015 and August 2024 were retrospectively analyzed. EAD was defined by the presence of one or more of the following criteria: total bilirubin ≥ 10 mg/dL or international normalized ratio ≥ 1.6 on postoperative day 7, and alanine or aspartate aminotransferase levels > 2000 IU/L within the first 7 days. *Results*: EAD occurred in 20 patients (21.5%). Patients with EAD exhibited significantly lower graft survival (*p* < 0.01) and patient survival (*p* = 0.03) compared with those without EAD. EAD was an in-dependent risk factor for both graft survival (*p* = 0.021) and patient survival (*p* = 0.027). Acute liver failure (odds ratio [OR], 6.228; 95% confidence interval [CI], 1.179–32.906; *p* = 0.031), donor age (OR, 1.051; 95% CI, 1.008–1.096; *p* = 0.020), and warm ischemic time (OR, 1.048; 95% CI, 1.001–1.098; *p* = 0.046) were identified as significant predictors of EAD development. *Conclusions*: EAD adversely affects both graft and patient survival following LT. Recipient clinical status, donor age, and intraoperative conditions should be carefully considered to minimize the risk of EAD.

## 1. Introduction

Liver transplantation (LT) remains the definitive treatment for patients with end-stage liver disease and selected cases of hepatocellular carcinoma (HCC) in cirrhotic livers [1]. Despite improvements in surgical techniques and perioperative care, graft insufficiency and failure continue to pose significant clinical challenges [2]. Early allograft dysfunction (EAD) refers to impaired initial graft function occurring shortly after LT [3,4,5]. The most widely accepted definition of EAD, proposed by Olthoff et al. [6] in a model for end-stage liver disease (MELD), is based on the presence of one or more of the following criteria: serum total bilirubin ≥ 10 mg/dL on postoperative day 7, international normalized ratio (INR) ≥ 1.6 on day 7, or aspartate aminotransferase (AST) or alanine ami-notransferase (ALT) > 2000 U/L within the first 7 days.

EAD is a relatively common complication, with an incidence rate ranging from 20% to 44%, and is associated with increased postoperative morbidity and mortality [7,8]. Although graft function may recover in some cases, approximately 7% of patients progress to graft failure, necessitating re-transplantation [9,10]. However, the feasibility of re-transplantation is limited by organ shortages, particularly in certain regions, thereby contributing to elevated mortality rates. Consequently, identifying risk factors for EAD is critical for guiding clinical decision-making during both donor selection and intraoperative management. Risk stratification may also facilitate individualized postoperative care and timely planning for potential re-transplantation, ultimately improving survival outcomes.

Several risk factors for EAD have been reported, including donor age, graft steatosis, donation after cardiac death (DCD), and perioperative variables such as cold ischemic time (CIT), warm ischemic time (WIT), and MELD score [5,11,12]. However, most of these studies have focused exclusively on deceased donor LT (DDLT). In contrast, living donor LT (LDLT) allows for the selection of healthier donors and grafts with reduced ischemia–reperfusion injury (IRI), achieved by minimizing CIT and WIT. Therefore, investigating EAD risk factors in a cohort that includes both DDLT and LDLT is warranted. Evaluating the impact of perioperative variables across a broader range of values may yield a more nuanced understanding of their roles in EAD development.

The objective of this study was to assess the impact of EAD on graft and patient survival and to identify associated risk factors in patients undergoing LT.

## 2. Patients and Methods

### 2.1. Patients

This retrospective study included 93 patients who underwent LT at our institution between July 2015 and August 2024, comprising 32 DDLTs and 61 LDLTs. Exclusion criteria were recipient age < 18 years, re-transplantation, and incomplete clinical data. EAD was defined by the presence of one or more of the following criteria: total bilirubin (TB) ≥ 10 mg/dL or INR ≥ 1.6 on postoperative day 7, and AST or ALT > 2000 IU/L within the first 7 days. Patients were stratified into two groups based on the presence of EAD: Group A (EAD, *n* = 20) and Group B (non-EAD, *n* = 73). Clinico-demographic characteristics and survival outcomes were compared between groups, and the effects of EAD on graft and patient survival were analyzed. In addition, perioperative risk factors associated with EAD development were assessed.

The study was approved by the Institutional Review Board of our institution (IRB No. 2407-001-19529) and was exempt from the requirement for informed consent due to the retrospective nature of the analysis and the absence of patient-identifiable data.

### 2.2. Data Collection

The following clinico-demographic and perioperative variables were collected: recipient age, sex, body mass index (BMI), presence of diabetes mellitus or hypertension, primary liver disease, MELD score, donor age, ABO incompatibility (ABOi), donor type (LDLT or DDLT), degree of hepatic macrosteatosis, operative time, CIT, WIT, and postoperative hospital stay. Laboratory values, including TB, INR, AST, and ALT, were recorded from admission through postoperative day 7. Graft failure was defined as re-transplantation or death. Follow-up duration was calculated from the date of LT to the last clinical visit or death.

### 2.3. Statistical Analysis

Normality of data distribution was assessed using the Shapiro–Wilk test. Normally distributed variables were expressed as means ± standard deviations and compared be-tween groups using Student’s *t*-test or the Kruskal–Wallis test, as appropriate. Categorical variables were compared using the χ^2^ test or Fisher’s exact test. Graft and patient survival were analyzed using the Kaplan–Meier method and compared with the log-rank test. Risk factors for graft and overall survival were assessed by multivariate analysis using the Cox proportional hazards model. Risk factors for EAD development were evaluated using multivariate logistic regression. Continuous variables identified as significant predictors were further analyzed using fractional polynomial regression. Statistical analyses were performed using SPSS Statistics for Windows, version 19.0 (IBM Corp., Armonk, NY, USA).

## 3. Results

### 3.1. Clinico-Pathologic Characteristics of Patients

The clinico-pathologic characteristics of the patients are summarized in Table 1. No significant differences were observed between groups in terms of age (53.6 ± 8.8 vs. 54.1 ± 11.9 years, *p* = 0.852), sex (65.0% vs. 68.5%, *p* = 0.767), prevalence of diabetes mellitus (70.0% vs. 69.9%, *p* = 0.991) or hypertension (80.0% vs. 78.1%, *p* = 0.853), ABOi transplantation (10.0% vs. 11.0%, *p* = 0.902), DDLT (50.0% vs. 30.1%, *p* = 0.098), hepatic steatosis > 10% (10.0% vs. 8.2%, *p* = 0.801), operative time (460 [225—815] vs. 475 [285—830] min, *p* = 0.523), or postoperative hospital stay (52.2 ± 73.2 vs. 38.8 ± 43.5 days, *p* = 0.305). Regarding primary liver disease, group A showed higher proportions of hepatitis B virus infection (25.0% vs. 13.7%), alcohol-related liver disease (45.0% vs. 42.5%), and acute liver failure (ALF; 25.0% vs. 4.1%), whereas group B showed higher proportions of hepatitis C virus infection (0% vs. 4.1%), hepatocellular carcinoma (5.0% vs. 27.4%), and cholestatic disease (0% vs. 8.2%). These distributions significantly differed between groups (*p* = 0.010). Laboratory MELD scores (29.5 [6—40] vs. 15 [6—40], *p* = 0.027), donor age (49.6 ± 16.5 vs. 39.2 ± 13.3 years, *p* = 0.005), CIT (3.5 ± 2.7 vs. 2.3 ± 2.0 h, *p* = 0.030), and WIT (48.6 ± 31.8 vs. 37.3 ± 8.8 min, *p* = 0.008) were significantly higher in group A than in group B. The peak levels of AST (3614 ± 5192 vs. 424 ± 336, *p* = 0.000) and ALT (1847 ± 2179 vs. 357 ± 274, *p* = 0.000) within the first 7days after LT were both significantly higher in group A than in group B. In general, the peak AST (91.8%) and ALT (75.0%) levels were recorded on the first day after LT.

### 3.2. Cumulative Probability of Graft and Overall Survival

**Figure 1:**
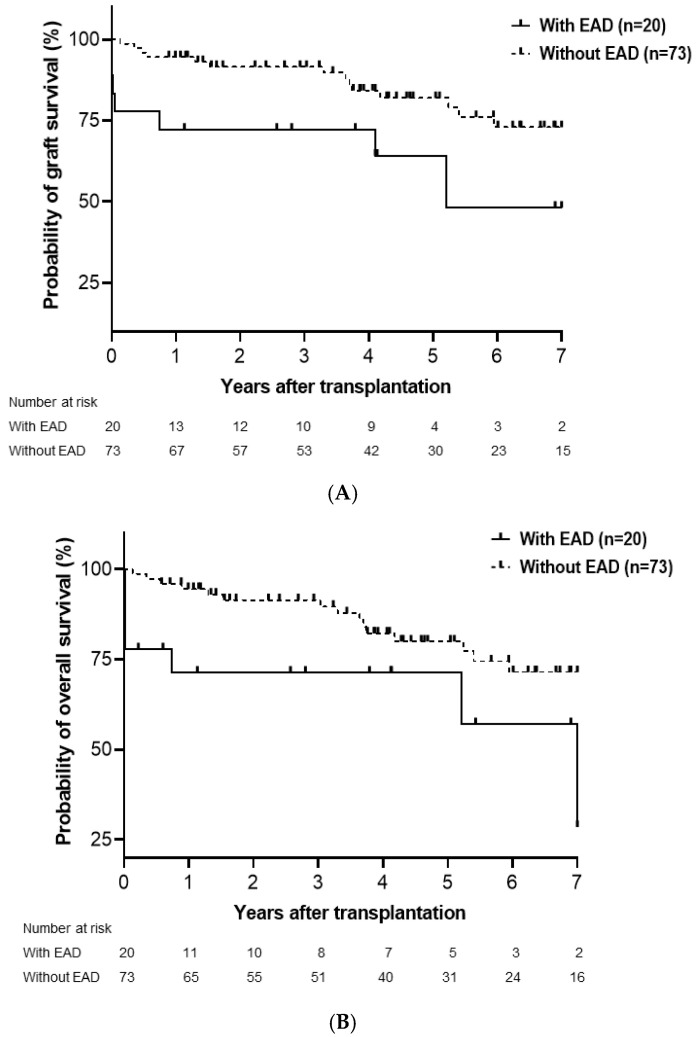
Cumulative probability of graft survival (**A**) and overall survival (**B**). Graft survival (*p* = 0.003) and overall survival (*p* = 0.003) were significantly lower in patients with early allograft dysfunction (EAD) compared with those without EAD. EAD, early allograft dysfunction.

The median follow-up duration was 4.1 (range, 0–8.8) years. The cumulative probability of graft survival significantly differed between groups based on the presence of EAD. In group A, 1-, 3-, and 5-year graft survival rates were 65.0%, 65.0%, and 57.8%, respectively, compared with 94.5%, 91.5%, and 82.0% in group B (*p* = 0.003; Figure 1A). Similarly, overall survival at 1, 3, and 5 years was 64.2%, 64.2%, and 64.2% in group A, versus 94.5%, 91.4%, and 80.0% in group B, with a statistically significant difference (*p* = 0.003; Figure 1B).

### 3.3. Cumulative Probability of Graft and Overall Survival in LDLT and DDLT

**Figure 2:**
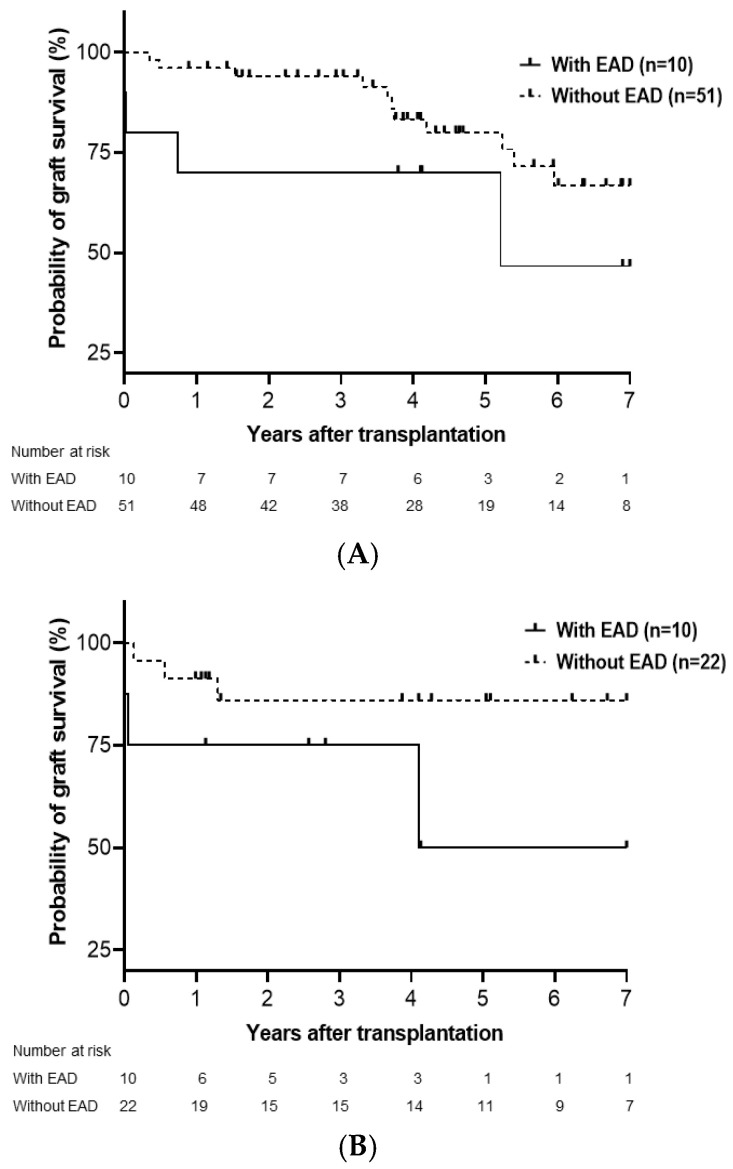
Cumulative probability of graft survival in LDLT (**A**) and DDLT (**B**). Graft survival was significantly lower in patients with EAD than in those without EAD in DDLT (*p* = 0.012) but not LDLT (*p* = 0.158). LDLT, living donor liver transplantation; DDLT, deceased donor liver transplantation; EAD, early allograft dysfunction.

**Figure 3:**
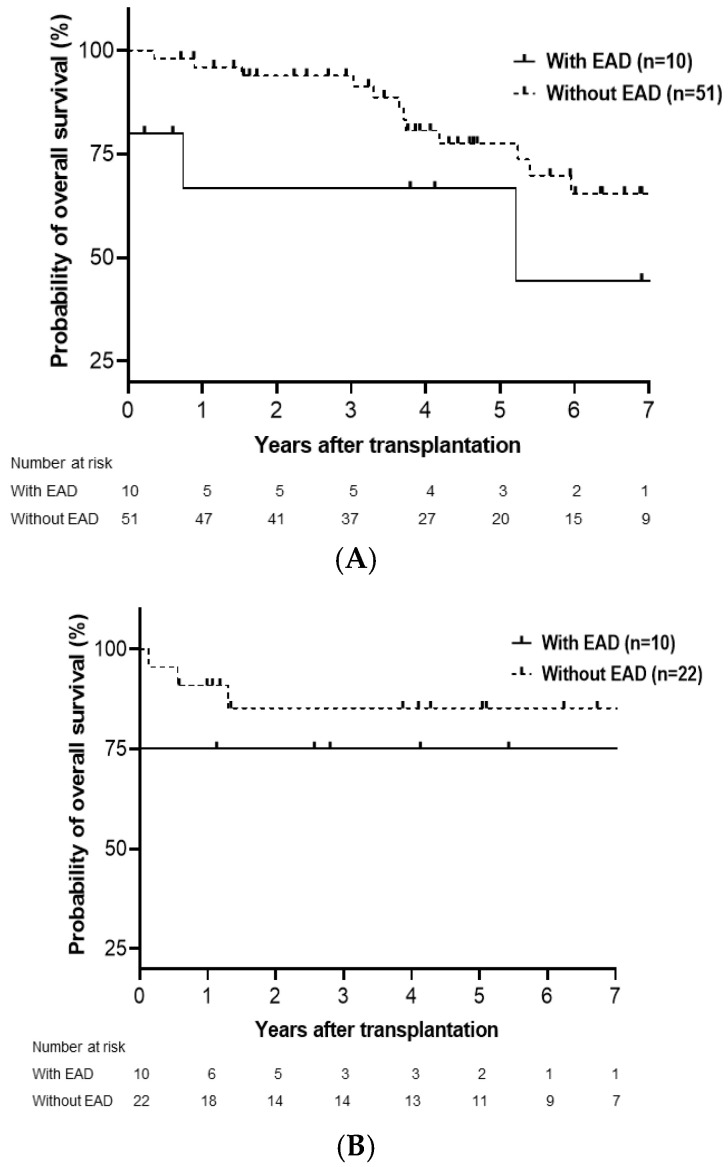
Cumulative probability of overall survival in LDLT (**A**) and DDLT (**B**). Overall survival in LDLT (*p* = 0.026) and DDLT (*p* = 0.033) was significantly lower in patients with EAD than in those without EAD. LDLT, living donor liver transplantation; DDLT, deceased donor liver transplantation; EAD, early allograft dysfunction.

Subgroup analysis of the cumulative probability of graft and overall survival in LDLT and DDLT was performed. Overall, graft survival was significantly influenced by EAD in DDLT (*p* = 0.012; Figure 2B) but not in LDLT (*p* = 0.158; Figure 2A). The overall survival significantly differed between patients with and without EAD in both LDLT (*p* = 0.026; Figure 3A) and DDLT (*p* = 0.033; Figure 3B) groups.

### 3.4. Risk Factor Analysis for Graft and Overall Survival

Univariate analysis identified WIT (hazard ratio [HR], 1.024; 95% confidence interval [CI], 1.006–1.042; *p* = 0.010) and EAD (HR, 3.388; 95% CI, 1.457–7.881; *p* = 0.005) as significant risk factors for graft survival. In multivariate analysis, EAD remained an independent predictor (HR, 2.866; 95% CI, 1.169–7.025; *p* = 0.021) (Table 2). For overall survival, univariate analysis revealed WIT (HR, 1.025; 95% CI, 1.009–1.041; *p* = 0.002) and EAD (HR, 3.329; 95% CI, 1.450–7.645; *p* = 0.005) as significant predictors. Both WIT (HR, 1.018; 95% CI, 1.001–1.035; *p* = 0.038) and EAD (HR, 2.728; 95% CI, 1.119–6.654; *p* = 0.027) remained significant in multivariate analysis (Table 3).

### 3.5. Risk Factor Analysis for EAD

**Figure 4:**
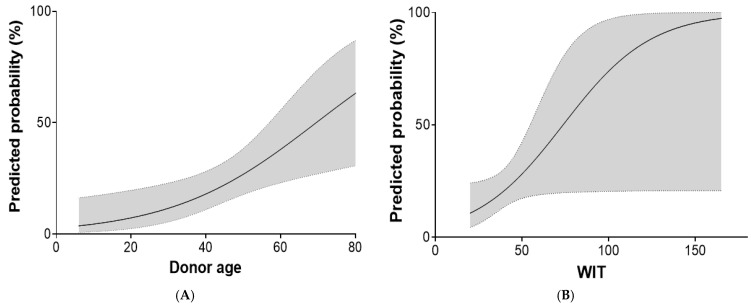
Association of donor age (**A**) and warm ischemic time (WIT) (**B**) with the risk of early allograft dysfunction (EAD). Curves were fitted using fractional polynomial regression; 95% confidence intervals are shown as gray shading.

Univariate analysis identified ALF (odds ratio [OR], 7.778; 95% CI, 1.674–36.141; *p* = 0.009), laboratory MELD score (OR, 1.045; 95% CI, 1.004–1.088; *p* = 0.031), donor age (OR, 1.053; 95% CI, 1.014–1.093; *p* = 0.008), and CIT (OR, 1.257; 95% CI, 1.037–1.557; *p* = 0.037) as significant risk factors for EAD. In multivariate analysis, ALF (OR, 6.228; 95% CI, 1.179–32.906; *p* = 0.031), donor age (OR, 1.051; 95% CI, 1.008–1.096; *p* = 0.020), and WIT (OR, 1.048; 95% CI, 1.001–1.098; *p* = 0.046) remained significant (Table 4). The associations of donor age (Figure 4A) and WIT (Figure 4B) with EAD are shown graphically.

## 4. Discussion

This study demonstrated that EAD was associated with significantly reduced graft and overall survival following LT, and it was identified as an independent risk factor for both outcomes. ALF, donor age, and WIT emerged as significant predictors of EAD.

EAD is a relatively common complication, with an incidence of 21.5% in this cohort. Its development has a profound impact on graft function and patient survival, often necessitating re-transplantation [8,13]. Patients who develop EAD have higher rates of sepsis, bleeding, and need for reoperation/re-transplantation. In our institution, intensive postoperative supportive management strategies are performed in this patient group, including optimizing hemodynamics with fluid resuscitation, correcting coagulopathy, administering empirical antibiotics for infections, and providing organ support including vasopressors or continuous renal replacement therapy if clinically indicated. Re-transplantation is indicated if patients have a high risk of early allograft failure. Therapeutic plasma exchange has been employed in some cases to enhance liver function by reducing bilirubin levels and improving coagulation parameters [14]. However, in severe cases, EAD is associated with high mortality unless graft recovery or re-transplantation is achieved, highlighting the importance of identifying risk factors to guide perioperative management and improve post-LT outcomes.

Donor characteristics substantially influence the risk of EAD. Previous studies have consistently shown that older donor age and higher BMI are associated with increased EAD risk [6,12,15,16,17]. In line with these findings, our results identified donor age as a significant risk factor. One study specifically reported a notable increase in EAD incidence with donor age exceeding 45 years [6], likely due to reduced hepatic progenitor cell populations and diminished regenerative capacity in older livers [12]. Although higher BMI has been linked to increased susceptibility to ischemia–reperfusion injury, often due to hepatic steatosis [17], our study did not find a significant association between BMI and EAD. This may be attributable to our institutional policy of excluding grafts with >30% macrovesicular steatosis from DDLT. Extended criteria donors, including donation after circulatory death or those with pre-existing hepatic injury, are also at elevated risk for EAD [18]. In LDLT, smaller graft size and low graft-to-recipient weight ratio (GRWR) are significant predictors of EAD, with elevated portal pressure implicated as a contributing factor [11,12]. In the present study, EAD development was not statistically significant in patients with a GRWR < 0.8, compared to that in others undergoing LDLT (22.2% vs. 15.4%, *p* = 0.609). All donors with a GRWR of <0.8 were young adults without liver steatosis. In our institution, we carefully assess the graft condition in marginal donors before proceeding with LDLT. This may explain the relatively better survival outcomes of LDLT in this study. The recipient’s preoperative condition is another critical determinant of EAD risk. A high MELD score has previously been identified as an independent predictor of EAD [19,20], reflecting the severity of underlying liver disease and associated systemic complications that may impair early graft function. Although MELD score was not significant in our analysis, ALF was a notable risk factor. ALF is marked by rapid hepatic decompensation, often accompanied by systemic inflammation and multi-organ failure. The persistence of this inflammatory milieu after LT likely contributes to the development of multi-organ failure, resulting in a need for EAD [21]. Additionally, a high visceral fat area has been reported as an independent risk factor for EAD, likely due to its pro-inflammatory and metabolically dysregulating effects [22].

Intraoperative variables also play a role in EAD development. CIT, when prolonged, is strongly associated with IRI, which compromises early graft function and elevates EAD risk [5,23]. However, in our cohort, all grafts had CIT < 6 h, and no significant association was observed. Recent studies have shown that normothermic machine perfusion improves graft viability and reduces EAD incidence compared to static cold storage [13]. WIT, by contrast, was significantly associated with EAD in our study. WIT contributes to direct hepatocellular injury from IRI, particularly under metabolically demanding warm conditions. This injury may be further exacerbated by increased intraoperative blood loss and transfusion requirements. Prior research has identified WIT > 45 min as a significant risk factor for EAD [11]. Moreover, surgical technique can influence WIT: the piggyback technique for hepatic vein reconstruction has been associated with shorter WIT compared to the bicaval technique [24].

Subgroup analysis revealed that EAD status resulted in a significant difference in graft survival in patients who underwent DDLT and those who underwent LDLT. When performing LDLT, we were able to select the donor in consideration of the graft condition and medical risks and benefits of the procedure for both the living donor and recipient. Donor age > 60 years, a remnant liver volume of <30%, or a GRWR of <0.8 were all considered as extended criteria resulting in further assessment of the graft condition. Living donors with >10% hepatic steatosis were administered a scheduled protein-rich diet and advised to perform exercises; after several weeks of body weight reduction, we re-analyzed the patients to confirm an improvement in their hepatic steatosis before surgery. These processes ensured that the graft quality of LDLT was generally superior to that of DDLT, which may have influenced the differences in graft failure in patients undergoing EAD after LT. Two patients who underwent LDLT and four who underwent DDLT experienced graft failure within 30 days after transplantation in this study. The two patients who underwent LDLT ultimately died due to sepsis and could not undergo re-transplantation. In the DDLT group, four patients had acute liver failure, which was identified as a risk factor for EAD in this study; of these patients, only one underwent re-transplantation and survived. It is important to consider donor age and graft quality to prevent EAD; normothermic machine perfusion can be used in selective cases. Further, the veno-veno bypass for the piggyback technique under stable vital signs during operation was considered to shorten the WIT. Re-transplantation would also be required in patients with a high risk of EAD after LT. Overall survival between patients with and without EAD was significantly for both LDLT and DDLT groups, indicating that the prevention of EAD is still important.

There are several limitations to this study. Its retrospective design means that the ac-curacy of the analyses depended on the completeness of hospital medical records. Additionally, the relatively small sample size limits the generalizability of the findings; larger, prospective studies are warranted to better elucidate the impact of EAD on clinical out-comes and to validate associated risk factors.

## 5. Conclusions

EAD was associated with significantly reduced graft and patient survival following LT. Acute liver failure, advanced donor age, and prolonged warm ischemic time were significant risk factors for EAD development. Patients presenting with these risk factors should be carefully managed before and during LT, with consideration given to early intervention strategies, including timely preparation for re-transplantation, to optimize clinical outcomes.

## Figures and Tables

**Table 1:** Clinico-demographic findings.

	With EAD (*n* = 20)	Without EAD (*n* = 73)	*p*
Age, years	53.6 ± 8.8	54.1 ± 11.9	0.852
Sex (male)	13 (65.0%)	50 (68.5%)	0.767
BMI	24.5 ± 4.2	23.6 ± 4.0	0.358
Diabetes mellitus	14 (70.0%)	51 (69.9%)	0.991
Hypertension	16 (80.0%)	57 (78.1%)	0.853
Original liver disease			
HBV	5 (25.0%)	10 (13.7%)	
HCV	0	3 (4.1%)	
Alcoholic	9 (45.0%)	31 (42.5%)	
HCC	1 (5.0%)	20 (27.4%)	
Cholestatic	0	6 (8.2%)	
Acute liver failure	5 (25.0%)	3 (4.1%)	0.010
Laboratory MELD	29.5 (6–40)	15 (6–40)	0.027
Donor age, years	49.6 ± 16.5	39.2 ± 13.3	0.005
ABOi	2 (10.0%)	8 (11.0%)	0.902
DDLT	10 (50.0%)	22 (30.1%)	0.098
GRWR < 0.8	2 (10.0%)	7 (9.6%)	0.956
Hepatic steatosis > 10%	2 (10.0%)	6 (8.2%)	0.801
Operative time, min	460 (225–815)	475 (285–830)	0.523
CIT, h	3.5 ± 2.7	2.3 ± 2.0	0.030
WIT, min	48.6 ± 31.8	37.3 ± 8.8	0.008
Peak AST within 7 days, U/L	3614 ± 5192	424 ± 336	0.000
Peak ALT within 7 days, U/L	1847 ± 2179	357 ± 274	0.000
Postoperative hospital stay, days	52.2 ± 73.2	38.8 ± 43.5	0.305

**Table 2:** Risk factors analysis for graft survival.

	Univariate			Multivariate		
	HR	95% CI	*p*	HR	95% CI	*p*
Age, years	0.994	0.956−1.034	0.763			
Sex (male)	1.580	0.584−4.271	0.367			
BMI	0.982	0.885−1.091	0.740			
DM	1.541	0.571−4.160	0.393			
HTN	1.768	0.524−5.964	0.358			
Primary liver disease	1.064	0.823−1.375	0.636			
Acute hepatic failure	2.273	0.671−7.701	0.187			
Hepatocellular carcinoma	0.653	0.221−1.928	0.441			
Laboratory MELD	1.017	0.983−1.053	0.334			
Donor age, years	1.017	0.989−1.047	0.242			
ABOi	0.814	0.191−3.476	0.782			
DDLT	1.130	0.479−2.667	0.781			
Hepatic steatosis > 10%	0.426	0.057−3.158	0.403			
Operative time, min	0.999	0.996−1.003	0.716			
CIT, h	1.132	0.946−1.356	0.177			
WIT, min	1.024	1.006−1.042	0.010			
EAD	3.388	1.457−7.881	0.005	2.866	1.169−7.025	0.021

**Table 3:** Risk factors analysis for overall survival.

	Univariate			Multivariate		
	HR	95% CI	*p*	HR	95% CI	*p*
Age, years	1.016	0.976−1.058	0.433			
Sex (male)	2.182	0.745−6.392	0.155			
BMI	1.033	0.938−1.138	0.512			
DM	0.980	0.405−2.372	0.964			
HTN	1.235	0.421−3.626	0.701			
Primary liver disease	0.958	0.709−1.295	0.781			
Acute hepatic failure	2.785	0.821−9.444	0.100			
Hepatocellular carcinoma	1.018	0.403−2.573	0.969			
Laboratory MELD	1.006	0.971−1.041	0.755			
Donor age, years	1.017	0.989−1.045	0.232			
ABOi	0.892	0.209−3.802	0.877			
DDLT	0.853	0.353−2.061	0.724			
Hepatic steatosis > 10%	0.856	0.200−3.655	0.834			
Operative time, min	1.001	0.998−1.005	0.406			
CIT, h	1.033	0.862−1.237	0.726			
WIT, min	1.025	1.009−1.041	0.002	1.018	1.001−1.035	0.038
EAD	3.329	1.450−7.645	0.005	2.728	1.119−6.651	0.027

**Table 4:** Risk factor analysis for EAD.

	Univariate			Multivariate		
	HR	95% CI	*p*	HR	95% CI	*p*
Age, years	0.996	0.953−1.041	0.850			
Sex (male)	0.854	0.301−2.425	0.767			
BMI	1.059	0.938−1.196	0.355			
DM	1.007	0.342−2.961	0.991			
HTN	1.123	0.329−3.834	0.853			
Acute hepatic failure	7.778	1.674−36.141	0.009	6.228	1.179−32.906	0.031
Hepatocellular carcinoma	0.139	0.018−1.112	0.063			
Laboratory MELD	1.045	1.004−1.088	0.031			
Donor age, years	1.053	1.014−1.093	0.008	1.051	1.008−1.096	0.020
ABOi	0.903	0.176−4.631	0.903			
DDLT	2.318	0.845−6.359	0.102			
Hepatic steatosis > 10%	1.241	0.231−6.676	0.802			
Operative time, min	1.002	0.997−1.006	0.476			
CIT, h	1.257	0.037−1.557	0.037			
WIT, min	1.040	1.000−1.082	0.051	1.048	1.001−1.098	0.046

## Data Availability

Data are available from the authors upon request because of our institutional ethical restrictions.

## Abbreviations

The following abbreviations are used in this manuscript: ABOi, ABO-incompatible transplantation; ALF, acute liver failure; ASTS, American Society of Transplant Surgeons; CIT, cold ischemic time; DCD, donation after circulatory death; DDLT, deceased donor liver transplantation; EAD, early allograft dysfunction; GRWR, graft-to-recipient weight ratio; IRI, ischemia–reperfusion injury; LDLT, living donor liver transplantation; LT, liver transplantation; MELD, model for end-stage liver disease; TB, total bilirubin; WIT, warm ischemic time.
